# Supplementary material for: Impact of Measurement Imprecision on Genetic Association Studies of Cardiac Function
Source: medRxiv. 2023 Feb 18:2023.02.16.23286058. Preprint. [Version 1] doi: 10.1101/2023.02.16.23286058 (PMC9949184; doi:10.1101/2023.02.16.23286058)
Supplement: 1 [file NIHPP2023.02.16.23286058v1-supplement-1.pdf]

medRxiv preprint doi: https://doi.org/10.1101/2023.02.16.23286058; this version posted February 18, 2023. The copyright holder for this preprint (which was not certified by peer review) is the author/funder, who has granted medRxiv a license to display the preprint in perpetuity.
It is made available under a CC-BY 4.0 International license .

Supplemental Material

Supplementary Tables

Supplementary Table 1. Mapping between Gaussian Noise SD and MAE

SD	MAE	R2
0	0	1
1	0.797489	0.9788
2	1.594416	0.9199
3	2.386753	0.8371
4	3.183924	0.743
5	3.974958	0.6508
6	4.793956	0.5632
7	5.604129	0.4832
8	6.380848	0.4192
9	7.228321	0.3602
10	7.920860	0.3183

medRxiv preprint doi: https://doi.org/10.1101/2023.02.16.23286058; this version posted February 18, 2023. The copyright holder for this preprint (which was not certified by peer review) is the author/funder, who has granted medRxiv a license to display the preprint in perpetuity.
It is made available under a CC-BY 4.0 International license .

Supplementary Table 2. Metrics of genetic signal for each decrease in cohort size

Cohort decrease	SNP Accuracy	GWAS Sensitivity
**0%**	1.0	1.0
**10%**	0.8744	0.8
**20%**	0.8713	0.8
**30%**	0.3436	1.0
**40%**	0.1392	0.4
**50%**	0.0477	0.4
**60%**	0.0019	0.2

medRxiv preprint doi: https://doi.org/10.1101/2023.02.16.23286058; this version posted February 18, 2023. The copyright holder for this preprint (which was not certified by peer review) is the author/funder, who has granted medRxiv a license to display the preprint in perpetuity.
It is made available under a CC-BY 4.0 International license .

Supplementary Figures

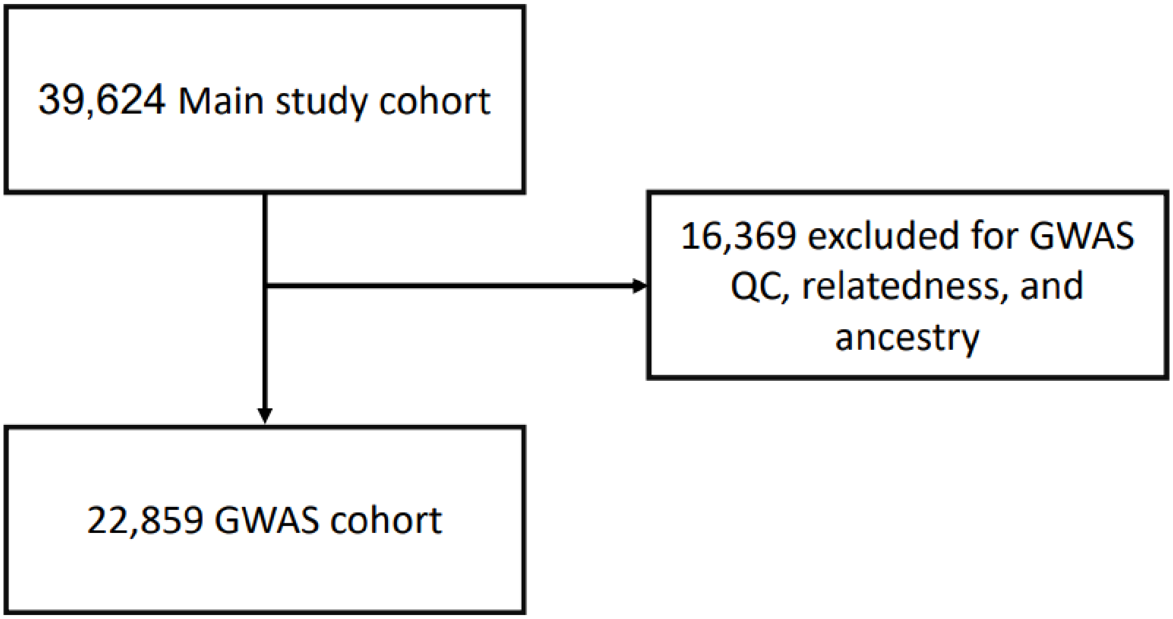

Supplementary Figure 1. Cohort diagram

medRxiv preprint doi: https://doi.org/10.1101/2023.02.16.23286058; this version posted February 18, 2023. The copyright holder for this preprint (which was not certified by peer review) is the author/funder, who has granted medRxiv a license to display the preprint in perpetuity.
It is made available under a CC-BY 4.0 International license .

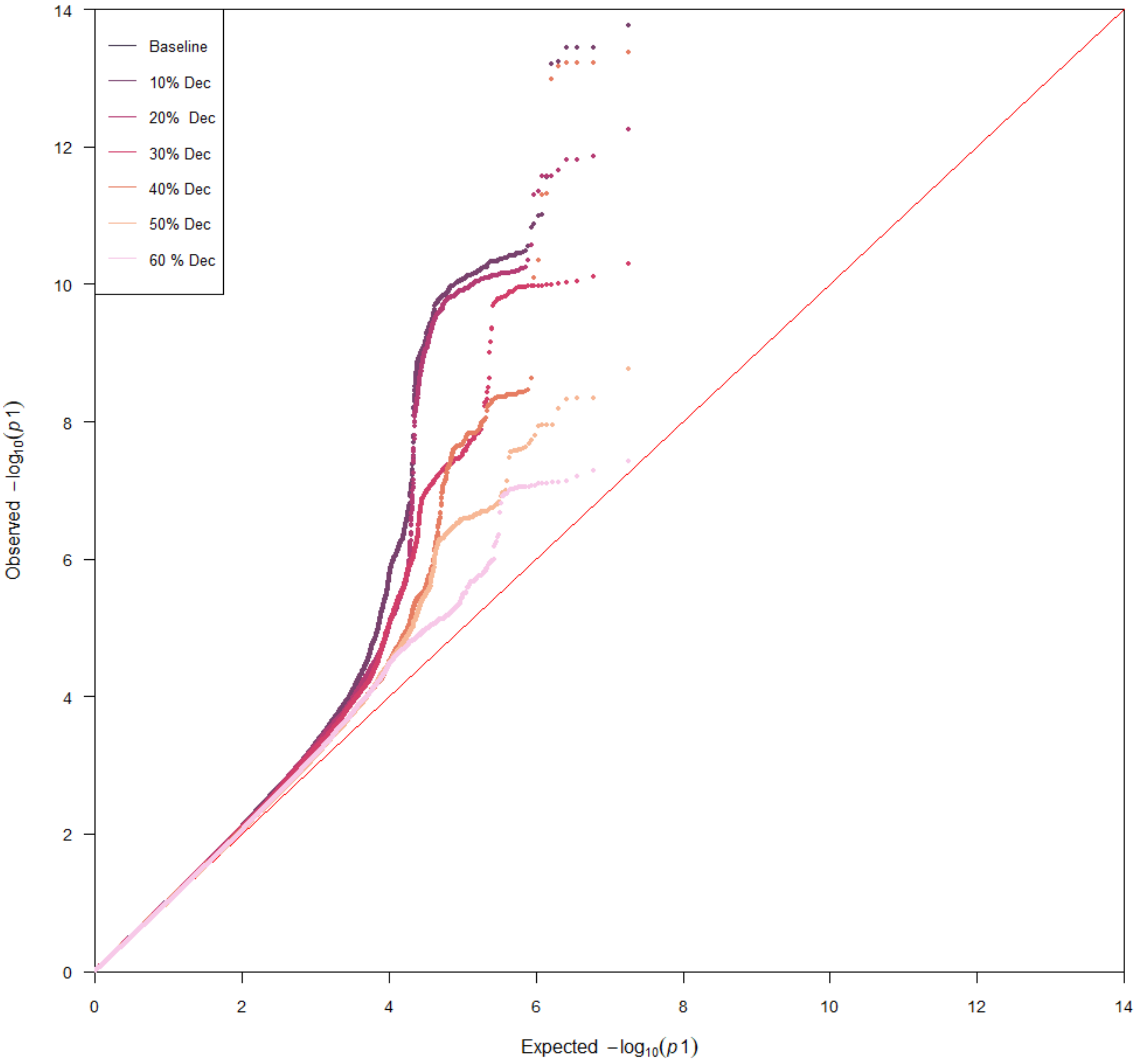

Supplementary Figure 2. *Q-Q plots of P values from GWAS summary statistics for different percentages of cohort decrease*
